# Association between numbers of decayed teeth and HbA1c in Japanese patients with type 2 diabetes mellitus

**DOI:** 10.1080/03009734.2017.1285838

**Published:** 2017-03-03

**Authors:** Satoru Yonekura, Masato Usui, Shunichi Murano

**Affiliations:** a Department of Endocrinology, Tochigi Medical Center, Shimotsuga, Ohira-machi Kawatsure 420-1, Tochigi City, Tochigi, Japan;; b Usui Dental Office, 12-14 Numawada-machi, Tochigi City, Tochigi, Japan

**Keywords:** Dental caries, diabetes, haemoglobin A1c, systemic disease

## Abstract

**Background:**

Dental caries (DC) are more prevalent in individuals with diabetes than in healthy individuals. However, the association between glycaemic control and DC has not been well characterized in patients with type 2 diabetes mellitus (T2DM). We therefore assessed the association between glycated haemoglobin (HbA1c) serum concentrations and the prevalence of DC in patients with T2DM.

**Methods:**

Retrospective analyses of data pertaining to 108 Japanese patients with T2DM hospitalized because of poor or worsened glycaemic control were included. We divided the patients based on HbA1c into two groups: HbA1c level ≥75 mmol/mol (9.0%) as poorly controlled T2DM, and HbA1c level <75 mmol/mol (9.0%) as a control group. We compared the association of lifestyle factors, dental caries, and periodontal health between patients with poorly controlled T2DM and controls. Stepwise multiple regression analyses were performed to evaluate the association between HbA1c, the absolute number of decayed teeth (DT), the sum of decayed, missing, and filled teeth, and the Met Need Index (MNI).

**Results:**

DT was higher and MNI was lower in patients with poorly controlled T2DM as compared to that in controls (*P* = 0.006 and *P* = 0.004, respectively). Multiple regression analyses revealed a significant association between HbA1c levels and DT (adjusted β = 0.039, 95% confidence interval [CI], 0.005 to 0.072, *P* = 0.025) and the MNI (adjusted β = −0.216, 95% CI −0.374 to −0.058, *P* = 0.008).

**Conclusions:**

DT and MNI were associated with HbA1c in T2DM patients.

## Introduction

Diabetes mellitus (DM) is a chronic metabolic disease characterized by impaired glycaemic control. The aetiology of type 2 diabetes mellitus (T2DM) includes both genetic and environmental factors (1). The condition is characterized by both impaired insulin secretion and peripheral insulin resistance (2). Uncontrolled DM may lead to dysfunction and failure of many organs, including the oral cavity (3).

Periodontal disease is a known complication of DM (4), which has an adverse effect on DM progression (5). In particular, periodontal disease-related tooth loss is significantly increased in DM patients relative to that in non-diabetic patients (6). DM patients tend to have difficulty maintaining good dietary habits necessary for obtaining good control of blood glucose, partly due to taste disturbances (7) and tooth loss (6).

Dental caries (DC) are caused by bacterial colonization on the tooth surfaces (8) and are known to lead to tooth loss (9). Currently available evidence on the association between DM and DC emanates largely from studies conducted in type 1 diabetes mellitus (T1DM) patients (10–17). Studies have shown a higher prevalence of DC in T2DM patients as compared to that in non-diabetic patients (18–21). However, no studies have compared the association of glycaemic control and DC in patients with well- or moderately controlled T2DM and poorly controlled T2DM.

The aim of this study was to determine whether higher glycated haemoglobin (HbA1c) levels are associated with a greater incidence of DC in poorly controlled T2DM patients as compared to that in well- or moderately controlled T2DM patients. We hypothesized that: 1) poorly controlled T2DM patients have higher numbers of decayed, missing, or filled teeth than would well- or moderately controlled T2DM patients; and 2) that the total number of decayed, missing, and filled teeth and the Met Need Index (MNI; an indication for treatment) are associated with the serum concentrations of HbA1c after adjusting for confounders in a multiple regression model.

## Materials and methods

### Study design and participants

**Figure 1.**
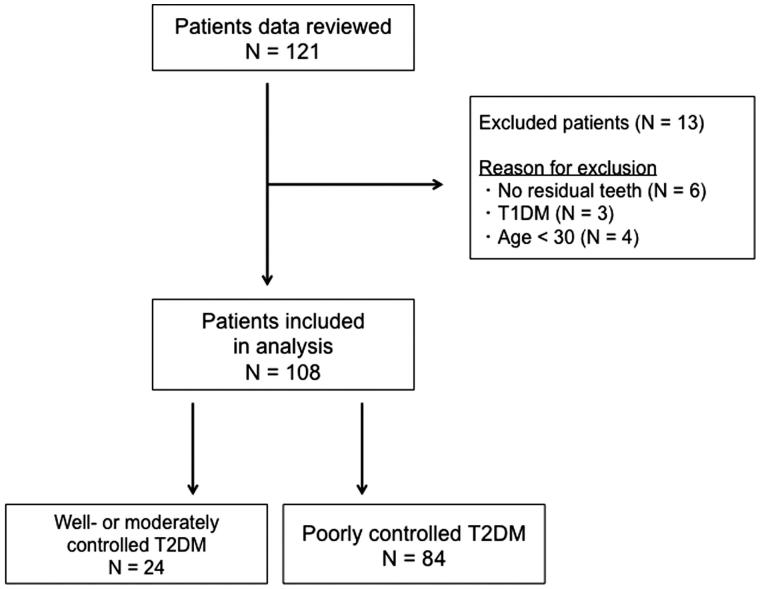
Study flow chart. T1DM: type I diabetes mellitus; T2DM: type 2 diabetes mellitus.

Data were retrospectively obtained from the records of 121 diabetic patients (71 male and 50 female patients), who were hospitalized for blood glucose control at the Tochigi Medical Center Shimotsuga, in Japan, in the period between January 2012 and August 2016. Their metabolic control was closely monitored, and it was tried to determine the cause of poor or worsened glycaemic control and to modify therapy (22). Patients having at least one remaining natural tooth were included in the analysis. Patients with T1DM, those on immunosuppressive therapy, acutely ill patients, and pregnant and lactating women were excluded. At the time of hospitalization, HbA1c concentrations were measured in all patients. Six patients (two men and four women) were excluded because they had no residual teeth. Three patients (two men and one woman) with T1DM were excluded. Four male patients were excluded because they were younger than 30 years (Figure 1), among whom were two teenagers. In children and adolescents, it remains unclear if HbA1c and the same HbA1c cut point should be used for T2DM diagnosis (23). A cut-off of HbA1c ≥75 mmol/mol (9.0%) was used to define poorly controlled T2DM patients, while HbA1c <75 mmol/mol (9.0%) was used to define well- or moderately controlled T2DM patients as a control group. A cut-off of HbA1c 75 mmol/mol (9.0%) is often used as a criterion to consider a shift from mono-therapy to a combination therapy (≥2 drugs) in order to achieve glycaemic control in clinical settings (24).

### Interview

All participants were interviewed using a standardized structured questionnaire. Three demographic variables were assessed: age, sex (male/female), and body mass index (BMI). Four lifestyle factors were assessed: alcohol consumption (current/former/none), smoking status (current/former/none), regular dental visits (yes/no), and regular exercise habits (yes/no). Age, sex, BMI, alcohol consumption, smoking status, and lack of regular exercise habits are known risk factors for T2DM (25).

### Oral examination and dental findings

During hospitalization, dentists clinically examined the dental status of all patients. All participants were informed regarding their dental diagnosis and offered appropriate dental treatment. Dental caries was measured visually without radiographs, and the numbers of decayed teeth (DT), missing teeth (MT), and filled teeth (FT) were recorded. Wisdom teeth were not considered. If a tooth had a decayed lesion at any site, it was considered decayed. Filled and crowned teeth were considered filled. Numbers were added to determine the DMF index as (DT + MT + FT). The DMF index generally reflects the lifetime caries experience of a person. To evaluate the caries experience between the poorly controlled T2DM patients and the control group, the absolute numbers of DT, FT, or MT were compared (15,26,27). The MNI was calculated for each individual as ([MT + FT]/[DT + MT + FT] × 100). The MNI is an indication of treatment received by an individual (28,29).

Bleeding on probing was considered positive if bleeding occurred within 30 seconds after probing. Periodontal probing depth was measured on each tooth; as periodontal probing depth ≥4 mm on at least one tooth has been shown to be associated with periodontitis (30), the number of teeth with periodontal probing depth ≥4 mm in depth was counted. The percentages of teeth with bleeding on probing and periodontal probing depth ≥4 mm, relative to the total number of teeth present, were obtained. The number of teeth with bleeding on probing and the number of teeth with periodontal probing depth ≥4 mm are indicators of periodontitis and risk factors for DC (31).

### Statistical analysis

Characteristics of patients with well- or moderately controlled T2DM or poorly controlled T2DM were compared. For continuous variables, Student’s *t* test for equal variances and Welch’s *t* test for unequal variances were used if a Shapiro–Wilk normality test showed a continuous variable to be normally distributed. For non-normally distributed continuous variables, a Mann–Whitney *U* test was used. Fisher’s exact test was performed for categorical variables.

We performed sensitivity analysis on the HbA1c cut-off values 64 mmol/mol (8.0%) and 69 mmol/mol (8.5%) as an index of poorly controlled T2DM in order to examine the effect of different HbA1c cut-offs on the results of the analyses. HbA1c cut-off values of 48 mmol/mol (6.5%), 53 mmol/mol (7.0%), and 59 mmol/mol (7.5%) were not used due to the small number of participants with those cut-off values (Supplementary table 1, available online).

To evaluate the association of the absolute number of decayed teeth, DMF index, and MNI with glycaemic control, we performed multiple linear regression analysis with stepwise variable selection based on the Akaike information criterion (AIC) (32). HbA1c was used as the main explanatory variable with adjustment for potential confounders: age, sex (male/female), BMI, smoking status (current/former/none), alcohol consumption (current/former/none), regular dental visits (yes/no), regular exercise habit (yes/no), duration of DM (years), percentage of teeth with bleeding on probing, and percentage of teeth with periodontal pocket depth ≥4 mm.

Multicollinearity was assessed using the variance inflation factor (VIF). A variance inflation factor >10 indicates serious multicollinearity, and values >4.0 may be a cause for concern. We assessed the VIF for all the covariates in each regression model, and none of the variables included had a VIF >4.0.

All *P* values were two-sided, and *P* values ≤0.05 were considered statistically significant. All statistical analyses were performed with EZR (Saitama Medical Center, Jichi Medical University, Saitama, Japan), which is a graphical user interface for R (The R Foundation for Statistical Computing, Vienna, Austria). More precisely, it is a modified version of R Commander designed to add statistical functions frequently used in biostatistics (33).

### Ethical approval

The present study was conducted in full accordance with the World Medical Association Declaration of Helsinki. The study protocol was reviewed and approved by the Ethics Committee at the Tochigi Medical Center Shimotsuga. The requirement for written informed consent was waived due to the retrospective nature of this study. All patient data were anonymized prior to the analysis.

## Results

### General characteristics

Baseline characteristics of poorly controlled T2DM (*n* = 84) and well- or moderately controlled T2DM (*n* = 24) are shown in Table 1. All patients were Japanese. Continuous variables were not normally distributed. Median age (range) of all participants was 59.0 years (33–88 years). The ratio of participants with poorly controlled T2DM and the control group was well balanced with regard to age, BMI, duration of DM, and all categorical variables. We performed sensitivity analyses by varying the HbA1c cut-off levels (64 mmol/mol [8.0%] and 69 mmol/mol [8.5%]). We observed similar results with respect to general characteristics (Supplementary tables 2, 3, 4 and 5, available online).

**Table 1.** Subject characteristics.

Characteristics	HbA1c <9.0 (*n* = 24)	HbA1c ≥9.0 (*n* = 84)	*P*^b^
Continuous variables^a^			
Age	64.5 (38–88)	59.0 (33–83)	0.102
BMI	25.52 (18.51–36.20)	26.20 (17.12–54.65)	0.671
Duration of DM (y)	9 (0.083–36)	5 (0.083–31)	0.242
Categorical variables			
Sex			
Male	15	48	0.815
Female	9	36	
Regular dental visits			
Yes	18	58	0.623
No	6	26	
Smoking status			
Current	6	23	0.329
Former	9	19	
None	9	42	
Alcohol consumption			
Current	4	8	0.529
Former	3	16	
None	17	60	
Regular exercise habit			
Yes	9	29	0.812
No	15	55	

a Continuous values are expressed as median (range).

b Continuous variables were subjected to Mann–Whitney *U* test. Categorical variables were subjected to Fisher’s exact test.

BMI: body mass index; DC: dental caries; DM: diabetes mellitus; HbA1c: glycated haemoglobin; T2DM: type 2 diabetes mellitus.

### Caries and periodontal status

Table 2 presents data regarding DC and periodontitis. All variables were non-normally distributed. There was no significant between-group difference with respect to percentage of teeth with bleeding on probing, percentage of teeth with periodontal pocket depth ≥4 mm, the numbers of filled or missing teeth, and DMF index. The median (absolute) number of decayed teeth was significantly higher, and the median MNI was significantly lower in poorly controlled T2DM patients as compared to the control group (*P* = 0.006 and *P* = 0.004, respectively). Sensitivity analyses using varying HbA1c cut-off levels revealed similar results on caries and periodontal status (Supplementary tables 2, 3, 4, and 5, available online), which indicated the robustness of the HbA1c cut-off criteria.

**Table 2.** Caries and periodontal status.

Characteristics^a^	HbA1c <9.0 (*n*: 24)	HbA1c ≥9.0 (*n*: 84)	*P*^b^
%BOP	23.21 (0–100)	32.74 (0–100)	0.150
%PPD ≥4 mm	18.33 (0–100)	31.07 (0–100)	0.325
DT	0 (0–11)	1 (0–19)	0.006
MT	2.5 (0–25)	3.5 (0–27)	0.558
FT	8.5 (0–24)	6.0 (0–26)	0.142
DMF index	17 (2–28)	17 (1–28)	0.909
MNI (%)	100 (50–100)	92 (5–100)	0.004

a All values are expressed as median (range).

b Continuous variables were subjected to Mann–Whitney *U* test.

%BOP: percentage of teeth with bleeding of probing; DMF: decayed, missing, and filled teeth; DT: number of decayed teeth; FT: number of filled teeth; HbA1c: glycated haemoglobin; MT: number of missing teeth; MNI: Met Need Index; %PPD ≥4 mm: percentage of teeth with periodontal probing depth ≥4 mm.

### Association between serum HbA1c and the absolute number of decayed teeth

Multiple linear regression model analyses with AIC-based stepwise variable selection demonstrated that the median (absolute) number of decayed teeth was significantly associated with HbA1c levels (adjusted β = 0.039, 95% confidence interval [CI] 0.005 to 0.072, *P* = 0.025) and having regular dental visits (adjusted β = −2.409, 95% CI −4.097 to 0.721, *P* = 0.006) (Table 3).

**Table 3.** Multiple linear regression analysis of exposure variables on the absolute number of decayed teeth.

	β	SE	95% LCI	95% UCI	*P* value
Constant	−2.054	2.635	−7.279	3.172	0.438
BMI	0.108	0.072	−0.035	0.251	0.137
Regular dental visits	−2.409	0.851	−4.097	−0.721	0.006
HbA1c (mmol/mol)	0.039	0.017	0.005	0.072	0.025

*n* = 108.

β: adjusted coefficient; HbA1c: glycated haemoglobin; LCI: lower value of a reliable interval; PPD: periodontal probing depth; %PPD: percentage of teeth with PPD ≥4 mm relative to the total number of teeth present; SE: standard error; UCI: upper value of a reliable interval.

### Association between HbA1c and DMF index

Multiple linear regression model analyses with AIC-based stepwise variable selection showed no association between DMF index and HbA1c levels (adjusted β = 0.061, 95% CI −0.001 to 0.122, *P* = 0.053) in T2DM patients (Table 4).

**Table 4.** Multiple linear regression analysis of exposure variables on the sum of decayed, missing, and filled teeth.

	β	SE	95% LCI	95% UCI	*P* value
Constant	2.164	5.018	−7.788	12.12	0.667
Age	0.157	0.066	0.027	0.288	0.018
Duration of DM	0.158	0.079	0.002	0.314	0.047
Regular exercise habit	−4.058	1.494	−7.021	−1.095	0.008
HbA1c (mmol/mol)	0.061	0.031	−0.001	0.123	0.053

*n* = 108.

β: adjusted coefficient; DM: diabetes mellitus; HbA1c: glycated haemoglobin; LCI: lower value of a reliable interval; SE: standard error; UCI: upper value of a reliable interval.

### Association between HbA1c and MNI

Multiple linear regression model analyses with AIC-based stepwise variable selection were used. The results revealed that MNI was significantly associated with HbA1c (adjusted β = −0.216, 95% CI −0.374 to −0.058, *P* = 0.008) and having regular dental visits (adjusted β = 12.09, 95% CI 4.150 to 20.02, *P* = 0.003) (Table 5).

**Table 5.** Multiple linear regression analysis of exposure variables on the Met Need Index.

	β	SE	95% LCI	95% UCI	*P* value
Constant	115.1	12.39	90.51	139.6	<0.01
BMI	−0.668	0.339	−1.339	0.004	0.051
Regular dental visits	12.09	4.002	4.150	20.02	0.003
HbA1c (mmol/mol)	−0.216	0.080	−0.374	−0.058	0.008

*n* = 108.

β: adjusted coefficient; HbA1c: glycated haemoglobin; LCI: lower value of a reliable interval; SE: standard error; UCI: upper value of a reliable interval.

## Discussion

The main objective of this study was to assess whether poor glycaemic control is associated with the prevalence of DC in T2DM patients. Our findings revealed the following: 1) poorly controlled T2DM patients were more likely to have a greater number of decayed teeth and lower MNI than were well- or moderately controlled T2DM patients; and 2) a higher number of decayed teeth and lower MNI were significantly associated with higher HbA1c in T2DM patients after adjusting for confounders.

Several mechanisms may explain the observed association between the absolute number of decayed teeth and HbA1c: *First*, poorly controlled T2DM patients may have more teeth with periodontitis, which can potentially promote DC. T2DM patients are at a greater risk of periodontitis (6) than non-diabetic patients. Periodontitis has been shown to be associated with DC (31). Therefore, DM-related periodontitis can potentially promote DC in T2DM patients. In the present study, however, periodontitis was not more common in poorly controlled patients, while more decayed teeth were observed in poorly controlled T2DM as compared to in the control group. In addition, percentage of teeth with bleeding on probing and percentage of teeth with periodontal pocket depth ≥4 mm were not associated with the absolute number of decayed teeth in the multiple regression model. Therefore, an association between DM-related periodontitis and DC was not observed in the present study. *Second*, poorly controlled T2DM patients have been shown to have a poor oral environment with cariogenic organisms such as *Treponema denticola*, *Prevotella nigrescens*, *Streptococcus sanguinis*, *Streptococcus oralis*, and *Streptococcus intermedius* in supragingival plaque (20) caused by hyposaliva (34), impaired salivary flow (35), upregulated salivary alkaline phosphatase activity, and lower salivary Ca^2+^ levels (36). And *third*, poorly controlled T2DM patients may lack appropriate awareness or habits for maintaining a good oral environment. DM patients are likely to have a lower frequency of brushing or flossing (37) and fewer dental visits (38) than non-diabetic patients. A study conducted in the USA with DM patients found that only 18.2% recognized that their oral health might be affected by diabetes (39). A firmer intention to brush is associated with lower HbA1c level, and a better dental attitude is known to be associated with better diabetic adherence (40).

In the present study there are no data on awareness of oral health risks caused by DM and on habits of brushing. We found that having regular dental visits was associated with fewer decayed teeth and greater MNI. Therefore, promotion of oral health awareness and encouraging T2DM patients both to brush their teeth regularly and to seek regular dental consultations may contribute to a lower prevalence of decayed teeth and improved glycaemic control in T2DM patients.

MNI assesses treated caries. In the present study, lower MNI was significantly associated with higher HbA1c. Therefore, it is more likely that the lower MNI in poorly controlled T2DM patients means that they have not had their caries treated to the same extent as the controls. This may be taken to indicate that dental intervention for decayed teeth may be beneficial for poorly controlled T2DM patients.

The current study has several limitations. *First*, the results of this hospital-based study may have been affected by selection bias introduced by the inclusion criteria. Inclusion of both in-patients and out-patients may help minimize selection bias. *Second*, specific sites of caries were not included in the analysis. The relationship between T2DM and DC may be different for coronal or root surface caries (21). *Third*, the current study may potentially overestimate the prevalence of DC because the DMF index in the present study may have included missing teeth that are not due to DC. *Fourth*, the current study may potentially underestimate periodontitis, as only bleeding on probing and periodontal pocket depth were used as indicators; other potential indicators include attachment loss, plaque index, gingival index, and alveolar bone resorption (41). *Fifth*, this study lacks data regarding daily diet, including the daily caloric or carbohydrate intake, awareness of oral health risk associated with DM, tooth brushing, use of toothpaste, or salivary biochemical and immunological profiles, all of which are liable to affect oral health. *Finally*, as this was a retrospective study, it does not provide evidence of a causal relationship between higher HbA1c and increased prevalence of decayed teeth in T2DM patients.

The present study suggests that higher HbA1c levels are associated with more decayed teeth and that there is a need for extended treatment of decayed teeth in T2DM patients. It also implies that it is necessary to promote amongst poorly controlled T2DM patients the awareness of the risks of tooth decay. To help T2DM patients maintain good oral health, physicians and dentists have to work together. It is then worthy of note that the use of dental care services as an entry-point for screening for DM and hypertension has been shown to be a cost-effective means for early detection of these conditions (42).

For further evaluation of a potential causal association between decayed teeth and T2DM, prospective cohort studies are needed with adjustment for confounders including demographics (age, sex, and BMI), lifestyle factors (smoking status, alcohol consumption, exercise habit, daily intake of glucose), oral health factors (tooth brushing habit, use of toothpaste, biochemical and immunological profile of saliva, and regular dental visits), dental examinations (caries sites, pocket depth, bleeding on probing, attachment loss, plaque index, gingival index, and alveolar bone resorption), and factors related to DM such as disease duration.

## Supplementary Material

Supplemental data
