# Supplementary material for: Association between numbers of decayed teeth and HbA1c in Japanese patients with type 2 diabetes mellitus
Source: Ups J Med Sci. 2017 Mar 3;122(2):108–13. doi: 10.1080/03009734.2017.1285838 (PMC5441370; doi:10.1080/03009734.2017.1285838)
Supplement: Supplemental data [file IUPS_A_1285838_SM3995.docx]

*Supplementary material for Yonekura S, et al. Association between numbers of decayed teeth and HbA1c in Japanese patients with type 2 diabetes mellitus, Upsala Journal of Medical Sciences, 2017*

**Supplementary materials**

**Supplementary table 1. Distribution of participants with different HbA1c cut-off values**

| HbA1c cut off | N with HbA1c < cut off | N with HbA1c ≥ cut off |
| --- | --- | --- |
| 9.0 | 24 | 84 |
| 8.5 | 16 | 92 |
| 8.0 | 6 | 102 |
| 7.5 | 1 | 107 |
| 7.0 | 0 | 108 |
| 6.5 | 0 | 108 |
|  |  |  |

HbA1c, glycated hemoglobin

**Supplementary table 2. Subject characteristics with HbA1c cut off of 8.5 for poorly controlled T2DM**

| Characteristics | HbA1c < 8.5 N = 16 | HbA1c ≥ 8.5 N = 92 | P† |
| --- | --- | --- | --- |
| Continuous variables* |  |  |  |
| Age | 64 (38–88) | 59 (33 - 83) | 0.279 |
| BMI | 25.42 (18.51–36.20) | 26.17 (17.12–54.65) | 0.914 |
| Duration of DM (yr) | 9.5 (0.083–35) | 5 (0.083–36) | 0.361 |
|  |  |  |  |
| Categorical variables |  |  |  |
| Sex |  |  |  |
| Male | 10 | 53 | 0.789 |
| Female | 6 | 39 |  |
| Regular dental visits |  |  |  |
| Yes | 12 | 64 | 0.773 |
| No | 4 | 28 |  |
| Smoking status |  |  |  |
| Current | 5 | 24 | 0.646 |
| Former | 5 | 23 |  |
| None | 6 | 45 |  |
| Alcohol consumption |  |  |  |
| Current | 4 | 8 | 0.179 |
| Former | 2 | 17 |  |
| None | 10 | 67 |  |
| Regular exercise habit |  |  |  |
| Yes | 8 | 30 | 0.256 |
| No | 8 | 62 |  |

* Continuous values are expressed as median (range)

† Continuous variables were subjected to Mann-Whitney U test. Categorical variables were subjected to Fisher's exact test.

HbA1c, glycated hemoglobin; T2DM, type 2 diabetes mellitus; DC, dental caries; BMI, body mass index.


**Supplementary table 3. Caries and periodontal status with HbA1c cut off of 8.5 for poorly controlled T2DM**

|  | HbA1c < 8.5  (N = 16) | HbA1c ≥ 8.5 (N = 92) | |
| --- | --- | --- | --- |
| Characteristics* |  |  | P† |
| %BOP | 32.74 (0–100) | 31.46 (0–100) | 0.587 |
| %PPD ≥4 mm | 22.96 (0–100) | 29.33 (0–100) | 0.635 |
| DT | 0 (0–9) | 1 (0–19) | 0.005 |
| MT | 3 (0–24) | 3 (0–27) | 0.817 |
| FT | 7 (0–17) | 7 (0–26) | 0.849 |
| DMF index | 15 (2–28) | 18.5 (1–28) | 0.090 |
| MNI (%) | 100 (57.14–100) | 92.58 (5–100) | 0.005 |

* All values are expressed as median (range).

† Continuous variables were subjected to Mann-Whitney U test.

HbA1c, glycated hemoglobin; BOP, bleeding of probing; PPD, periodontal probing depth; DT, decayed teeth; MT, missing teeth; FT, filled teeth; DMF, decayed, missing, and filled teeth; MNI, Met Need Index


**Supplementary table 4. Subject characteristics with HbA1c cut off of 8.0 for poorly controlled T2DM**

| Characteristics | HbA1c < 8.0 N = 6 | HbA1c ≥ 8.0 N = 102 | P† |
| --- | --- | --- | --- |
| Continuous variables* |  |  |  |
| Age | 65 (38–88) | 59 (33 - 83) | 0.274 |
| BMI | 22.63 (19.61–35.81) | 26.17 (17.12–54.65) | 0.331 |
| Duration of DM (yr) | 14.5 (1–29) | 5.5 (0.083–36) | 0.138 |
|  |  |  |  |
| Categorical variables |  |  |  |
| Sex |  |  |  |
| Male | 3 | 60 | 0.692 |
| Female | 3 | 42 |  |
| Regular dental visits |  |  |  |
| Yes | 5 | 71 | 0.667 |
| No | 1 | 31 |  |
| Smoking status |  |  |  |
| Current | 2 | 27 | 1.00 |
| Former | 1 | 27 |  |
| None | 3 | 48 |  |
| Alcohol consumption |  |  |  |
| Current | 1 | 11 | 0.804 |
| Former | 1 | 18 |  |
| None | 4 | 73 |  |
| Regular exercise habit |  |  |  |
| Yes | 4 | 34 | 0.181 |
| No | 2 | 68 |  |

* Continuous values are expressed as median (range)

† Continuous variables were subjected to Mann-Whitney U test. Categorical variables were subjected to Fisher's exact test.

HbA1c, glycated hemoglobin; T2DM, type 2 diabetes mellitus; DC, dental caries; BMI, body mass index.

**Supplementary table 5. Caries and periodontal status with HbA1c cut off of 8.0 for poorly controlled T2DM**

|  | HbA1c < 8.0  (N = 6) | HbA1c ≥ 8.0 (N = 102) | |
| --- | --- | --- | --- |
| Characteristics* |  |  | P† |
| %BOP | 0 (0–50) | 32.14 (0–100) | 0.095 |
| %PPD ≥4 mm | 1.613 (0–55.56) | 29.33 (0–100) | 0.074 |
| DT | 0 (0–0) | 1 (0–19) | 0.015 |
| MT | 4.5 (0–24) | 3 (0–27) | 0.951 |
| FT | 4.5 (0–14) | 7 (0–26) | 0.349 |
| DMF index | 13 (2–24) | 17.5 (1–28) | 0.098 |
| MNI (%) | 100 (100–100) | 93.10 (5–100) | 0.016 |

* All values are expressed as median (range).

† Continuous variables were subjected to Mann-Whitney U test.

HbA1c, glycated hemoglobin; BOP, bleeding of probing; PPD, periodontal probing depth; DT, decayed teeth; MT, missing teeth; FT, filled teeth; DMF, decayed, missing, and filled teeth; MNI, Met Need Index
